# Tumor mutation burden: from comprehensive mutational screening to the clinic

**DOI:** 10.1186/s12935-019-0929-4

**Published:** 2019-08-07

**Authors:** Francesca Galuppini, Carlo Alberto Dal Pozzo, Jutta Deckert, Fotios Loupakis, Matteo Fassan, Raffaele Baffa

**Affiliations:** 1 Department of Medicine, Surgical Pathology Unit, University of Padua, Via Aristide Gabelli, 61, 35121 Padua, Italy; 2 Servier Pharmaceuticals, Boston, MA USA; 3 Veneto Institute of Oncology, IOV-IRCCS, Oncology Unit 1, Padua, Italy

**Keywords:** Tumor mutation burden, Immunotherapy, Next generation sequencing, Target therapy

## Abstract

The recent advent of immunomodulatory therapies into the clinic has demanded the identification of innovative predictive biomarkers to identify patients most likely to respond to immunotherapy and support the design of tailored clinical trials. Current molecular testing for selection of patients with gastrointestinal or pulmonary carcinomas relies on the prevalence of PD-L1 expression in tumor as well as immune cells by immunohistochemistry and/or on the evaluation of the microsatellite status. Tumor Mutational Burden (TMB) has emerged as a promising novel biomarker in this setting to further aid in patient selection. This has been facilitated by the increasing implementation of molecular pathology laboratories with comprehensive next generation sequencing (NGS) technologies. However, the significant overall costs and expertise required for the interpretation of NGS data has limited TMB evaluation in routine diagnostics, so far. This review focuses on the current use of TMB analysis in the clinical setting in the context of immune checkpoint inhibitor therapies.

## Tumor mutational burden and checkpoint inhibition

Cancer has been described as a genomic disease driven by an accumulation of both germline derived and somatic mutations. Recent advances in molecular techniques have allowed the evaluation of the impact of germline and tumor mutational status on overall cancer risk, patient prognosis and treatment response. Tumor mutation frequency varies widely among cancer types and between tumors of the same histotype. Tumor Mutational Burden (TMB) can be analyzed by various methods and is reported as the total number of sequence variants or mutations per tumor genomic region analyzed [1].

High mutational burden is typical of cancers developed as a consequence of exposure to powerful carcinogens, such as tobacco smoke and polycyclic aromatic hydrocarbons in lung cancers and bladder cancers, as well as exposure to mutagens, such as ultraviolet light in melanoma.

**Fig. 1:**
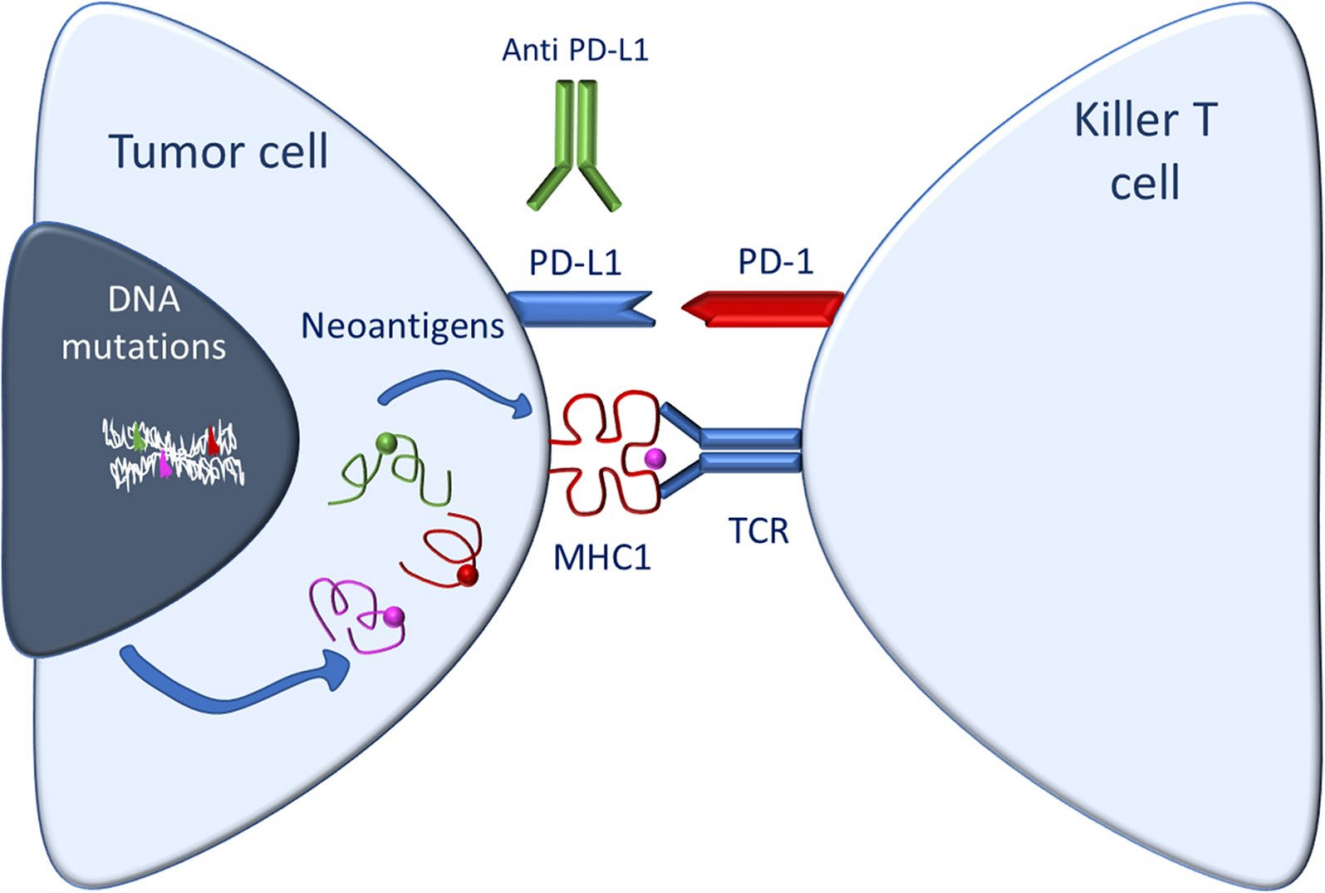
Schematic diagram of tumor cell with high TMB and its relationship with the immune system. The formation of neoantigens enhanced immune cell recognition and the effectiveness of immunotherapy

In recent years, the interest in TMB by physicians and researchers has increased as tumors with higher TMB can be more responsive to immune checkpoint inhibitor therapies, which may be due to their increased inherent immunogenicity [2]. An effective host anti-tumor immune response requires tumor cell surface antigen recognition followed by priming and activation of immune cells that can ultimately mediate tumor cell killing. However, inhibitory receptor interactions on immune cells are often hijacked by tumors to dampen cytotoxic T cell responses against transformed cells thereby avoiding immune surveillance. For example, programmed death ligand 1 (PD-L1) expressed on tumors engages the immune checkpoint PD-1 on cytotoxic T lymphocytes to block their action against tumor cells [3]. Likewise, Cytotoxic T lymphocyte antigen 4 (CTLA-4) engagement by B7-1 (CD80)/B7-2 (CD86) ligands constitutes another key inhibitory checkpoint signal that limits T-cell activation. Immune checkpoint modulating drugs aim to remove these inhibitory signals to boost the immune response against cancer cells and relieve innate as well as adaptive immune resistance developed by tumors. It has been suggested that patients who do not derive benefit from immune checkpoint inhibitor therapies lack pre-existing anti-tumor T-cell responses, in part due to low immunogenicity of their underlying disease [4–6]. Mutational load, and in particular, nonsynonymous mutations, in cancer cells may generate novel antigens (termed neoantigens) that are not subject to immune tolerance and allow for an adaptive immune response by the host (Fig. 1). The observation that nonsynonymous mutation burden is associated with efficacy of the anti-PD-1 antibody pembrolizumab is consistent with this hypothesis [3, 7]. Moreover, several preclinical [8–13] and clinical [14–17] reports have demonstrated that neoantigen-specific effector T cell response lies at the core of recognizing and eliminating established tumors.

It is however important to note that TMB alone does not represent a direct evidence of tumor immunogenicity, due of the complex dynamics that underlie the host immune response in the context of tumor cells and their microenvironment.

## Clinical impact of TMB

The translational significance of TMB assessment is derived from its link to tumor immunogenicity and its subsequent prognostic and predictive values. In fact, several recent studies have demonstrated that TMB can be exploited as a biomarker, especially in order to predict patient responses to immune checkpoint modulatory agents (Table 1).

**Table 1:** Representative recent studies describing the impact of tumor mutation load (TMB) evaluation in the clinical setting

Type of cancer and stage	No of investigated patients	Test for TMB	Cut-off	Drug/treatment	Results	References
SCLC	211 (133 Nivo, 78 Nivo + Ipi)	WES	≥ 248 total mut	Nivolumab, Nivolumab + Ipilimumab	Improved ORR, 1y PFS and 1y OS for TMB high vs. TMB medium and low patients	[21]
NSCLC	34 (16 discovery, 18 validation)	WES	≥ 178 total mut	Pembrolizumab	Higher TMB was associated with improved objective response, durable clinical benefit and PFS	[20]
NSCLC	312 (158 Nivo, 154 Chemo)	WES	≥ 243 total mut	Nivolumab vs chemotherapy	High TMB associated with increased ORR and PFS, but not OS	[22]
NSCLC	240	NGS (49 also with WES)		Anti-PD-(L)1 monotherapy or in combination with anti-CTL-4	Elevated TMB improved likelihood of benefit to ICIs Targeted NGS accurately estimates TMB and correlates with WES results	[30]
Stage IV or recurrent NSCLC	299, 139 (Nivo + Ipi), 160 (chemotherapy)	NGS		Nivolumab plus ipilimumab, vs. chemotherapy	PFS was longer with first line nivolumab plus ipilimumab than with chemotherapy and a high TMB, irrespective of PD-L1 expression level	[40]
Metastatic melanoma	65 (32 + 33)	NGS		Nivolumab or pembrolizumab or atezolimumab	Response rate, PFS and OS were superior in high mutation load group	[6] l
Metastatic melanoma	64 (25 discovery +39 validation)	WES	> 100 total mut	Ipilimumab or tremelimumab	Mutational load is associated with the degree of clinical benefit	[18]
CRC	6004	Comprehensive Genomic Profiling (CGP)		–	TMB classifies MSI tumors as TMB-high and identifies nearly 3% of CRC as MSS/TMB-high	[27]
CLL	91	WES		allo-HSCT	Clinically evident durable remission in patients with neoantigen peptides	[15]
26 cancer types	11,348	NGS and MSI-NGS		–	MSI offers distinct data for treatment decision regarding immune checkpoint inhibitors, in addition to TMB and PD-L1	[28]
12 cancer types	86	MSI-NGS		Pembrolizumab	Large proportion of mutant neoantigens in MSI cancers make them sensitive to immune checkpoint blockade, regardless the cancer’s tissue of origin	[29]

*NSCLC* Non-small cell lung cancer, *WES* whole-exome sequencing, *PFS* progression-free survival, *OS* overall survival, *allo-HSCT* allo-hematopoietic stem cell transplantation, *TILs* tumor-infiltrating lymphocytes, *MSI* microsatellite instability, *ICIs* immune checkpoint inhibitors

The development of drugs against well-described PD-1/PD-L1 and CTLA-4/B7 checkpoints interactions have proven effective in unleashing a cytotoxic response against malignant cells and have revolutionized the therapeutic approaches to various solid tumors, including those typically marked by strong resistance to traditional chemotherapeutics [4]. This is illustrated by the particular effectiveness of both nivolumab, and pembrolizumab (targeting PD-1) and ipilimumab (targeting CTLA-4) against a subset of patients with Non-Small Cell Lung Cancer (NSCLCs) and melanoma. Despite the significant benefit potentially achievable with these new therapies, response rates vary widely between cancer types and there is a particular need for predictive biomarkers that can be applied upfront to identify patients more likely to respond to immune checkpoint modulators. To this end, the predictive value of various features of tumors, host immune cells and the tumor microenvironment have been further explored including PD-L1 expression in both tumor and immune cells, selected single gene mutational status, peripheral-blood lymphocyte count, tumor infiltrating lymphocyte count, markers of T-cell activation and evaluation of inflammatory cytokines [18]. Nevertheless, none of these have been unambiguously associated with patient outcome endpoints including Overall Survival (OS), Progression Free Survival (PFS), and Objective Response Rate (ORR) across multiple tumor types. To date, microsatellite instability-high (MSI-H) status, mismatch repair deficiency and PD-L1 expression constitute the only predictive biomarkers successfully used for patient selection in select malignancies.

Since TMB assessment is not exempt from economical and technical issues, it is important to establish a list of malignancies that are more likely to be highly mutated and, thus, to be priority candidates for this analysis.

Here the etiology of the diverse neoplastic pathologies plays a critical role. In principle, TMB is likely to be high especially in two categories of tumors: (i) those that arise from the exposure to powerful carcinogenic and mutagenic agents (e.g. tobacco smoke and UV-A) and (ii) those caused by germline mutations in genes encoding for proteins involved in DNA repair and replication.

### TMB in lung cancer

The predictive role of TMB in pulmonary metastatic tumors constitutes a remarkable paradigm [19]. Indeed, immune checkpoint inhibitor therapies have proven effective against pulmonary malignancies, whose etiology is in most cases linked to exposure to the “carcinogenic cocktail” contained in cigarette smoking [19]. In fact, both TMB rate and effectiveness of immune checkpoint inhibitors are higher in neoplasms developed by smokers in comparison to the ones developed by never-smokers. This is likely a result of the “signature of smoking” [20] which is characterized by a transversion-high mutational profile that results in an increased number of non-synonymous mutations and, ultimately, in a greater neoantigens load. Furthermore, efficacy of immunomodulatory therapies in Small Cell Lung Cancer (SCLCs) appears to be lower than in NSCLCs, while SCLC is also marked by a lower number of mutations per megabase than NSCLCs.

A crucial study published in 2015 by Rizvi et al. [20] demonstrated that higher nonsynonymous mutation burden in tumors was associated with improved ORR, durable clinical benefit (DCB), and prolonged PFS in a retrospective analysis of two cohorts of NSCLC patients treated with the anti-PD-1 pembrolizumab. Furthermore, this analysis suggested that the efficacy of immune checkpoint inhibition by pembrolizumab in the treatment of NSCLC also correlated with the molecular smoking signature, higher neoantigen burden, and the presence of DNA repair pathway mutations, but not with patient HLA haplotype. Of note, the authors provided a TMB threshold (based on nonsynonymous mutation burden) suitable to identify candidate patients in clinical practice: in the validation cohort a cut-off of 178 mutations per tumor provide sensitivity of about 85% and specificity of about 75% in differentiating responders from non-responders.

Building on the positive predictive value of high TMB for the response to anti-PD1/PD-L1 and anti-CTLA-4, many subsequent analyses focused on identification of standardized cut-offs suitable to harmonize the results of various clinical trials and the relationships of TMB with other biomarkers, in particular PD-L1 expression.

A crucial analysis by Hellman and co-workers [21] on > 200 SCLC patient samples from the CheckMate 032 trial compared outcomes for patients treated with nivolumab or a combination of nivolumab and ipilimumab. Patients were grouped into tertiles based on TMB analysis and this analysis revealed improved ORR and favorable 1-year PFS and OS outcomes for TMB-high (≥ 248 mutations) patients treated with nivolumab or a combination of nivolumab and ipilimumab compared to patients with medium or low TMB. In contrast, PD-L1 expression status was not predictive of response and no association was found between PD-L1 expression and TMB.

In the Phase 3 CheckMate 026 trial in first-line PD-L1 positive NSCLC patients, nivolumab treatment was not associated with improved ORR or prolonged PFS versus chemotherapy. However, an exploratory analysis of 312 patients evaluated the effect of TMB on outcomes, with patients divided into tertiles based on TMB status. ORR and PFS were improved in the TMB-high group (≥ 243 mutations) treated with nivolumab as compared to chemotherapy, while OS was similar [22]. Again, PD-L1 expression was not predictive of response, even for a subset with PD-L1 ≥ 50%, and did not correlate with TMB.

Similarly, in a subgroup analysis of the Phase 3 CheckMate 227 trial on ~ 300 metastatic NSCLC patients with TMB of > 10 mutations/Mb, combination treatment with Nivolumab and Ipilimumab resulted in prolonged PFS compared to chemotherapy treatment [40]. Importantly, in this study TMB was an independent positive predictive biomarker, irrespective of tumor PD-L1 expression level. The value of TMB assessment was also confirmed in a multivariate analysis including sex, tumor histologic type and ECOG performance-status. Therefore, the authors suggested that combined therapy with nivolumab plus ipilimumab may represent an effective first-line treatment regimen for patients with advanced NSCLCs marked by high TMB, irrespective of PD-L1 expression level and other investigated clinical and pathological variables. Unfortunately, final analysis of this trial population is still pending, making it uncertain if TMB status will achieve regulatory approval as a predictive biomarker for this treatment regimen in metastatic NSCLC.

In another study, 240 NSCLC patients treated with anti-PD-1 or anti-PD-L1 based therapy were profiled for TMB by targeted NGS. TMB was significantly associated with improved patient outcomes with the increased odds of disease control with increasing thresholds [30]. Importantly, comparison of NGS with WES analysis showed high correlation between these methods.

### TMB in melanoma

The etiology of melanoma is typically related to the mutagenic effects of UV exposure. Comprehensive sequencing data (i.e. 507 whole genome and 6535 whole exome sequences) from the International Cancer Genome Consortium (ICGC) pinpointed malignant melanoma as the tumor with the highest mutation prevalence among 27 different histotypes [23]. The genotoxic effects of the UV-A are comparable to those of smoking. Studies of Weber et al. (including patients with melanoma who have progressed after being treated with ipilimumab and BRAF inhibitors) [24] and Robert et al. [25] (including metastatic melanoma patients, negative for BRAF mutation, not treated previously) reported that checkpoint inhibitors have been particularly successful in melanoma, with the highest response rates to single-agent PD-1/PD-L1 inhibition. In this indication, the connection between TMB and tumor immunogenicity is again highlighted by the fact that non-cutaneous melanomas have far fewer mutations than those of cutaneous origin, and show are less responsive to immunotherapy.

In an early report from 2014, Snyder et al. [18] found that high mutational load correlates with a sustained clinical benefit from CTLA-4 blockade in patients suffering from metastatic melanomas. Nonetheless, high TMB alone is clearly not sufficient for clinical benefit, as not all tumors with a high mutational burden responded to therapy. This study further identifies the existence of specific tumoral neoantigens that create a neoepitope signature as critical for the response to anti-CTLA-4 and overall high mutational load increased the probability of such a signature being present in melanoma patients.

Another retrospective study reported by Johnson et al. [6] evaluated the predictive role of TMB in two cohorts of patients with metastatic melanoma treated with anti-PD-1/PD-L1 antibodies. In both cohorts ORR, PFS and overall survival (OS) was superior in the high, compared to intermediate and low mutation load groups. This is the first demonstration of TMB as an independent prognostic value for multiple immune checkpoints-modulators. Moreover, the authors introduced a stratified evaluation of TMB, which could represent a practical strategy to overcome the limits of a single threshold value in terms of sensitivity and specificity.

### TMB in other cancers

A study by Powles et al. [26] investigated outcomes of metastatic platinum-refractory urothelial carcinoma patients treated with atezolizumab versus chemotherapy in the IMvigor211 trial. Here, high PD-L1 expression was not a predictive biomarker. However, in an exploratory analysis in > 500 samples, high TMB correlated with increased OS in patients treated with atezolizumab, supporting the role of TMB as an alternate biomarker.

In addition, CRC presents a perfect opportunity to study the role of DNA-repair deficiency in the development of high TMB. In an elegant study by Fabrizio et al. [27] TMB assessment has been connected to microsatellite status and DNA-repair genes mutational status in a cohort of patients with metastatic CRCs. Here almost all (301/302, 99.7%) MSI-high patients were classified as TMB-high and their tumors were marked by mutations in DNA-repair genes such as *MSH2*, *MSH6* and *MLH1*. This suggests that MSI-high status could serve as a surrogate biomarker of high TMB in metastatic CRCs in order to predict response to immune checkpoints modulation therapy. However, MSI-high status alone may not capture all metastatic CRCs potentially sensitive to anti-PD1/PD-L1 drugs. In fact, in the same study MSI-high tumors accounted for only for 97% of all TMB-high metastatic CRCs. Thus, an estimated 3% of high-TMB metastatic CRC tumors are microsatellite stable (MSS). Due to the high prevalence and mortality related to metastatic CRC (about 50,000 deaths/year), the correct identification of TMB-high/MSS malignancies might lead to a substantial therapeutic benefit for an additional 1500 patients/year. These considerations underline once again the critical predictive value of comprehensive TMB assessment, which is cannot be easily replaced by other surrogate biomarkers.

Therefore, the importance of TMB, microsatellite and PD-L1 status and the interplay between them differs significantly between cancer types which may be context and mechanism dependent. Thus further investigations are needed to better understand their respective translational value alone and in combination [28]. In the future, this will ideally enable more precise patient stratification based on assessment of multiple biomarkers, preferably from the same patient biopsy.

## Testing TMB: the technical approach

The gold standard and most widely used method to assess TMB for research purposes is whole exome sequencing (WES). Indeed, many of the studies cited above have used WES as the basis for the retrospective TMB evaluation. However, this technique is expensive and requires excessively lengthy turnaround times making it unsuitable for large scale and routine clinical applications [2]. To overcome this problem, two alternative approaches have been proposed: (i) the development of “surrogate biomarkers” that could be easily evaluated; (ii) the indirect determination of TMB by evaluation of the mutational status of a defined gene panel by Next Generation Sequencing (NGS).

The intrinsic shortcoming of the first proposal is that any surrogate biomarker would represent an approximation for TMB, which in itself is a representation of tumor immunogenicity. Any indirect TMB assessment will likely introduce an even greater margin for error. Overall, biomarkers suitable for this purpose include MSI status [27, 29] (see above), chromosomal structural analyses [19] and mutational analyses of selected genes. The latter approach might be useful in specific tumor subtypes, despite the existence of the abovementioned intrinsic issues. For example, metastatic melanomas bearing mutations in *LRP1B* gene have a significantly higher mutational load as compared with *LRP1B* wild type tumors; furthermore, mutational load correlates with the number of *LRP1B* mutations *per* tumor [6]. This is likely due to the size and chromosomal location of *LRP1B*. Indeed, this is a large putative tumor suppressor gene, located in a common fragile chromosomal site. Due to these unique structural characteristics, *LRP1B* mutational status is likely a good approximation of total exonal mutational load. Other genes whose mutational status might be associated with high TMB are *KRAS* in NSCLCs (due to its link to the smoking signature), *MSH2*, *MSH6*, *MLH1* in CRCs (due to the relationship between MSI and high TMB) [27, 28] and finally *NF1*, *BRAF*, and *NRAS* in metastatic melanomas [6].

In contrast, NGS appears to be a more powerful tool to translate TMB assessment in clinical practice. Many studies [2, 4, 19, 28, 30] have demonstrated that TMB evaluation by comprehensive genomic profiling on NGS diagnostic platforms correlated well with gold-standard WES evaluation. This approach can provide several notable advantages: (i) NGS is available in many academic centers and it has already become commonplace in clinical oncology [2]; (ii) NGS is less expensive than WES; (iii) NGS analysis shortens the assay turnaround times to < 10 days; which is the recommended timeframe for clinical decision making on the basis of molecular characterization of neoplasms [31]; (iv) in addition to TMB, NGS can also provide information on other critical prognostic and predictive factors (e.g. *EGFR*, *KRAS*, and *BRAF* in NSCLCs or *KRAS*, *NRAS* and *BRAF* in CRCs). In addition, the simultaneous assessment of multiple actionable genomic targets in the gene panel [32, 33] may help to further refine the molecular determinants of response to immunotherapy, going beyond the “simple” assessment of TMB.

## TMB assessment: open issues

While it is now apparent that TMB is a valuable biomarker to predict the response to PD-1/PD-L1 and CTLA4/B7-1 axes inhibitors, there are still several problems that impede the routine adoption of this predictive approach. These barriers reside in the analysis itself as well as the pre- and post-analytic phases.

Widespread implementation of TMB testing in the clinic is still challenging. Sample size, sample quality and the resulting DNA yields are rate-limiting factors from the patient biopsy perspective. Cost associated with testing, including the need for specialized equipment and highly-trained personnel, can further limit the implementation of the TMB analysis in a routine setting. In addition, varying testing platforms, different bioinformatic pipelines, and non-standardized cut-off definitions hinder comparison between data sets from different sites and studies described in the literature. It is apparent that standardization of TMB evaluation is needed in order to ensure reliability, reproducibility and clinical utility [34].

Size of the NGS gene panel is critical for accurate analysis. It has been shown that as NGS panels for TMB analysis become smaller in size, the uncertainty associated with TMB estimation increases rapidly. Specifically, the coefficient of variance increases rapidly when the size of the targeted panels is less than 1 Mb [35]. Therefore, the minimum size of the panel for determining the TMB of more than 300 genes or 1 Mb has been proposed.

Scoring of detected alterations has not been standardized. Although the higher occurrence of synonymous variants may indicate a mutational process that also results in nonsynonymous changes, synonymous and germline variants are commonly discarded in the calculation of TMB, as it is assumed that these variants are unlikely directly involved in neoantigen generation. In the setting of tumor-only sequencing, germline false positive variants may be filtered out by using large and available germline variant data sets. The use of these germline databases is a critical step in this process, and it is necessary to use germline databases with a sufficiently broad representation of all populations. The variability of the TMB among different studies is due also to different scoring of alterations, as some studies consider all alterations including the copy number alterations [30], while others exclude copy number changes [36], variants included in COSMIC or alterations that are likely to be or are known to be *bona fide* oncogenic drivers and germline polymorphisms [37] in order to avoid bias.

In clinical practice, the testing of mutational burden should be performed early during the decision-making process in order to select the most appropriate first-line treatment. Thus, a sufficient quantity and quality of tumor samples is required [21, 38]. Nevertheless, biopsy collection can be difficult as patients who are candidates for immunotherapy often suffer from metastatic disease by definition and often present with poor performance status (PS). As a consequence, not all patients will have sufficient tumor tissue available or will be able to safely undergo a biopsy. Therefore, less invasive sampling methods are warranted. In this perspective, the study of circulating tumor DNA could provide a non-invasive method for assessing TMB, but further research is needed to establish the performances of this approach in clinical settings.

While the ongoing debate about the best method for TMB evaluation favors NSG over WES and evaluation of surrogate biomarkers for technical and practical reasons, standards have not been established for gene panels used in NSG approaches. In addition, implementation of an organizational framework for the next generation surgical pathology laboratories is crucial, in order to maximize the impact of TMB assessment on clinical decision making. Current guidelines recommend a timeframe of 3 workdays from a request for testing to receipt by a reference laboratory and 10 workdays for availability of testing results [32, 33, 39]. According to observation of VanderLaan et al. [31] in their institution (Beth Israel Deaconess Medical Center Boston, MA) these objectives were reached in only 50% of cases when a comprehensive NGS assay was performed. These findings demonstrate that a turnaround time of 10 workdays is indeed technically feasible with the current available technologies, since half of the samples has been successfully analyzed within that period. Yet, they also highlight that reliable testing workstreams have not been established and while the ability to comply with current guidelines might differ between various centers, multidisciplinary efforts are needed in order to continuously improve turnaround times. These should include efficient workflows for automated ordering of molecular profiling, investment in infrastructure to expedite data delivery, and improved software to streamline somatic variant analysis [31].

Once TMB data have been successfully collected, the main bottleneck is their interpretations. The main problems derive from (i) the intrinsic complexity of biological meaning of TMB; (ii) the lack of an unambiguous key to understand the data, and (iii) the multiple therapeutic protocols put in place for highly-mutated malignancies.

With regard to the first issue, it has been already mentioned that the translational value of TMB assessment lies in its assumed relationship with tumor immunogenicity. Nevertheless, immunogenicity is overall a multifactorial feature, which is only partially due to the mutational load. TMB alone cannot accurately assess the dynamic immune status of both tumor and tumor microenvironment. Therefore, it is rational to assume that the comprehensive and integrated evaluation of genomic (TMB, single gene mutations, MSI etc.) [27], immunohistochemical (e.g. PD-L1 expression) [19, 21] and histological (e.g. tumor grading and tumor infiltrating lymphocytes—TILs) [19] variables can significantly refine the selection of patients candidate for treatment with immune-checkpoints-modulators.

Another major obstacle to make TMB assessment clinically meaningful is the lack of a widely approved cut-off score (and an associated NGS panel) which must be set to successfully establish TMB as a predictive biomarker. To date, no agreement has been reached between the various authors. Some authors prefer a single threshold value to select patients for the administration of immune-checkpoint-modulators. For example, in the cohorts studied by Rizvi et al. using WES [2] a load of at least 178 mutations per exome provided a sensitivity of 86% and a specificity of 75% in identifying patients who can benefit from immunotherapy. Studies applying NGS an select gene panels with approximately 200–300 genes have either categorized TMB simply as high (at or above the median) or low (less than the median) without specifying a discreet cut-off value [26, 39] or have set a single cut-off [27, 33]. In many studies, malignancies have been further stratified in several classes according to their mutational load (e.g. low/intermediate/high TMB) [6, 19]. This approach has the virtue of allowing a greater level of therapeutic personalization in the future although this is yet untested. For example, patients with high TMB may be more likely to respond to a single-drug immunomodulatory treatment, while patients bearing an intermediated mutational load could benefit from more aggressive but potentially more toxic combination regimens (e.g. anti-PD-L1 combined with anti CTLA4 antibodies).

Finally, even if we were able to efficiently determinate and interpret TMB predictive value, there are still important open issues in the choice of best immunomodulatory treatment for each patient. Unaddressed questions concern the role of single agent treatments compared with combination treatments, the potential association between immunotherapy and chemotherapy and the preferred sequencing of therapies. Furthermore, there is an open question of how TMB analysis impacts the risk–benefit balance in patients receiving immune checkpoint inhibitors. The study by Hellmann [40] evaluated efficacy outcomes for several treatment regimens (co-primary endpoint PFS with Nivolumab + Ipilimumab vs Chemotherapy in patients with TMB ≥ 10 mut/Mb, and as secondary endpoint PFS with Nivolumab vs Chemotherapy in patients with TMB ≥ 13 mut/Mb and ≥ 1% PD-L1 expression). Treatment-related adverse events resulting in discontinuation and serious adverse event rates were higher for the nivolumab + ipilimumab combination than chemotherapy in all treated patients and the subgroup with TMB ≥ 10 mut/Mb. Yet, this combination treatment also resulted in improved PFS in the TMB-high population.

## Conclusions

This review of current available literature suggests that TMB is a robust predictive biomarker to select patients for immune checkpoint immunotherapy that could be readily applied in many metastatic cancer settings. This assessment promises to be particularly useful for malignancies developed as a consequence of the exposure to powerful mutagens and carcinogens (e.g. NSCLCs, SCLCs and melanomas) or marked by frequent mutations in genes involved in DNA repair and replication (e.g. MSI-high CRCs). In these indications TMB should be considered as a reliable tissue-based biomarker for immune checkpoint blockade. Several comprehensive studies performed in recent years have provided evidence for a link between TMB and patient outcomes in NSCLCs and melanomas, which are among the most highly mutated human tumors. Subset analysi of TMB-high patients in other malignancies have supported the same observation.

TMB has clearly emerged as a robust predictive biomarker, yet there is still debate about its value by itself rather than in combination with other variables, such as PD-L1 expression, TILs and critical/driver single-gene mutations. Furthermore, there are remaining logistical issues that hamper implementing TMB evaluation in daily clinical practice. These include difficulties in obtaining sufficient biopsies from often critically ill patients as well as lack of a standard analysis method that is economically viable and combines robust performance with an acceptable turnaround time. In this respect, the use of NGS on selected-gene panels emerges as the best tool currently available to more widely implement TMB assessment in clinical practice. Nevertheless, large-sized randomized trials are warranted to provide and confirm standardized cut-offs required to confirm TMB assessment as a meaningful predictor of patient outcomes. Additional characterization of tumor biology such as single-gene mutational status, MSI status and presence of TILs should not be considered as a substitute for TMB assessment, but can rather complement it to better predict individual patient response to immunotherapy. This is underscored by the observation that in NSCLC TMB is an independent predictive biomarker, irrespective of PD-L1 expression [40]. Applying our knowledge of tumor-subtype etiology may improve the predictive value of TMB assessment by identifying the diseases that are a priori more likely to bear high mutational load.

Based on the literature data, wide spread application of TMB analysis in clinical practice is still premature and hurdles remain for routine implementation. However, emerging data from large randomized trials currently ongoing may provide more definite criteria for its interpretation and demonstrate its clinical feasibility.

## Data Availability

Not applicable.
